# Supplementary material for: Bacteria Associated with the Roots of Common Bean (Phaseolus vulgaris L.) at Different Development Stages: Diversity and Plant Growth Promotion
Source: Microorganisms. 2022 Dec 24;11(1):57. doi: 10.3390/microorganisms11010057 (PMC9861878; doi:10.3390/microorganisms11010057)
Supplement: Supplementary file 1 [file microorganisms-11-00057-s001.zip › Table S1.pdf]

Genera	V1			V2			F			P			Total
	Nodules (N)	In (In)	Out (Out)	Nodules (N)	In (In)	Out (Out)	Nodules (N)	In (In)	Out (Out)	Nodules (N)	In (In)	Out (Out)	
***Pseudomonas* sp.**	2	2	5	4	5	2	3	-	8	3	6	3	43
***Enterobacter* sp.**	4	4	7	1	5	3	-	1	1	-	-	-	26
Bacillus sp.	2	-	3	-	1	1	-	2	5	-	6	4	24
Flavobacterium sp.	3	-	5	3	1	2	1	2	3	-	-	2	22
***Priestia* sp.**	-	-	2	1	-	1	-	2	1	-	5	2	14
***Kosakonia* sp.**	-	-	-	2	6	2	-	-	-	-	-	-	10
***Variovorax* sp.**	-	1	-	-	-	-	1	1	-	1	2	2	8
***Delftia* sp.**	-	2	-	-	1	2	-	-	2	-	-	-	7
***Achromobacter* sp.**	-	1	-	-	1	-	2	-	-	-	1	-	5
***Chitinophaga* sp.**	-	-	-	-	1	1	1	1	-	-	-	-	4
***Microbacterium* sp.**	-	-	-	-	-	-	-	-	-	-	2	2	4
***Rhizobium* sp.**	-	-	-	2	-	-	-	-	-	-	-	1	3
***Leclercia* sp.**	1	1	1	-	-	-	-	-	-	-	-	-	3
***Curtobacterium* sp.**	-	-	-	-	-	2	-	-	-	-	-	-	2
***Stenotrophomonas* sp.**	-	1	1	-	-	-	-	-	-	-	-	-	2
***Mucilaginobacter* sp.**	-	-	-	-	-	-	-	-	1	1	-	-	2
***Flexibacter* sp.**	-	-	-	-	1	-	-	-	-	-	-	-	1
***Azospirillum* sp.**	-	-	-	-	-	1	-	-	-	-	-	-	1
***Trinickia* sp.**	-	-	-	-	-	1	-	-	-	-	-	-	1
***Cronobacter* sp.**	-	-	-	-	-	1	-	-	-	-	-	-	1
***Pantoea* sp.**	1	-	-	-	-	-	-	-	-	-	-	-	1
***Klebsiella* sp.**	1	-	-	-	-	-	-	-	-	-	-	-	1
***Acidovorax* sp.**	-	-	-	-	-	-	-	-	-	1	-	-	1
***Cupriavidus* sp.**	-	-	-	-	-	-	-	-	-	-	1	-	1
***Agrobacterium* sp.**	-	-	-	-	-	-	-	-	-	-	-	1	1
***Paraburkholderia* sp.**	-	-	-	-	-	-	-	-	-	-	-	1	1
**Total**	14	12	24	13	22	19	8	9	21	6	23	18	189
