# Supplementary figures and images for: Bacteria Associated with the Roots of Common Bean (Phaseolus vulgaris L.) at Different Development Stages: Diversity and Plant Growth Promotion
Source: Microorganisms. 2022 Dec 24;11(1):57. doi: 10.3390/microorganisms11010057 (PMC9861878; doi:10.3390/microorganisms11010057)

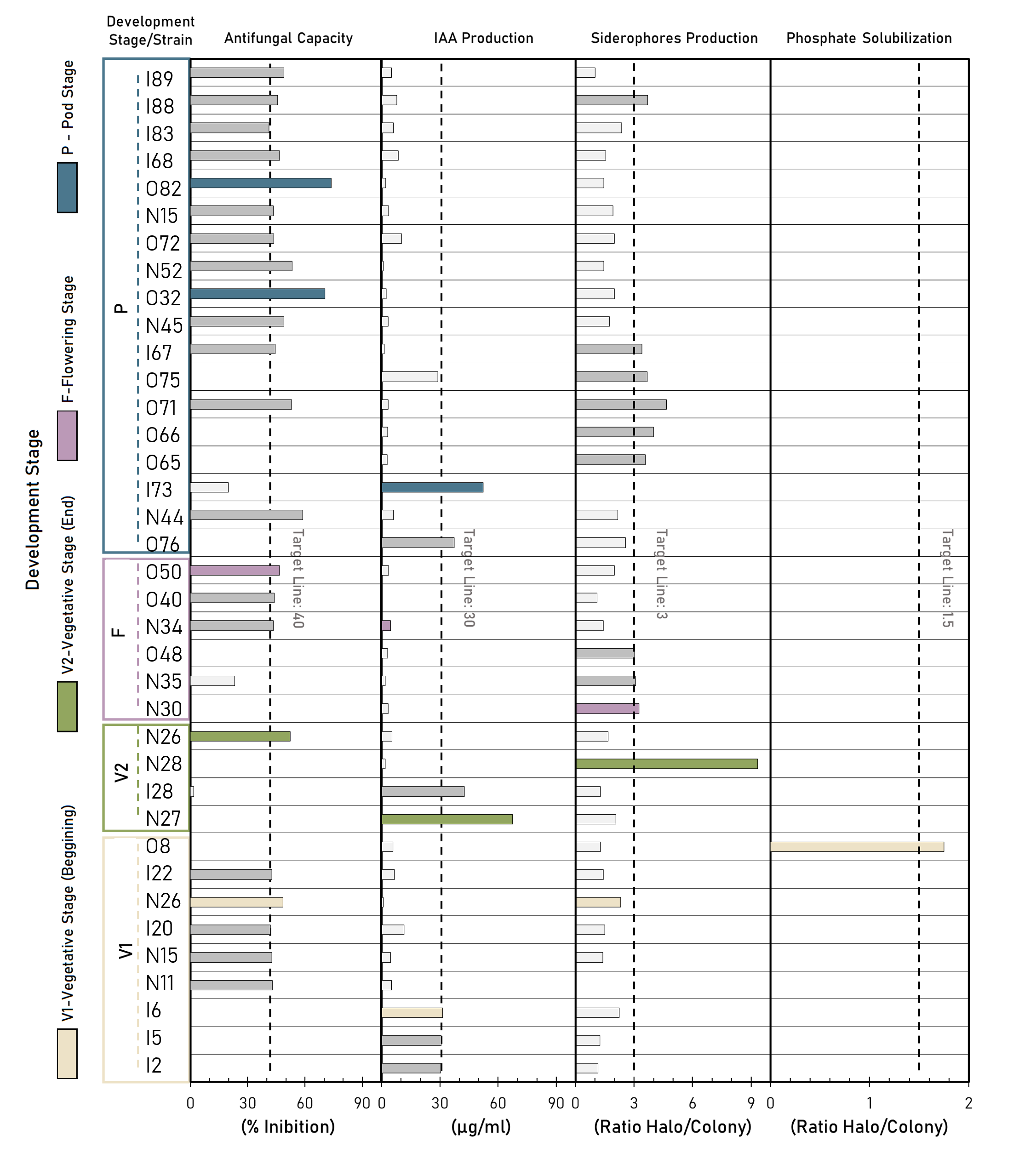

Supplement: Supplementary file 1 [file microorganisms-11-00057-s001.zip › Figure S1.tif]

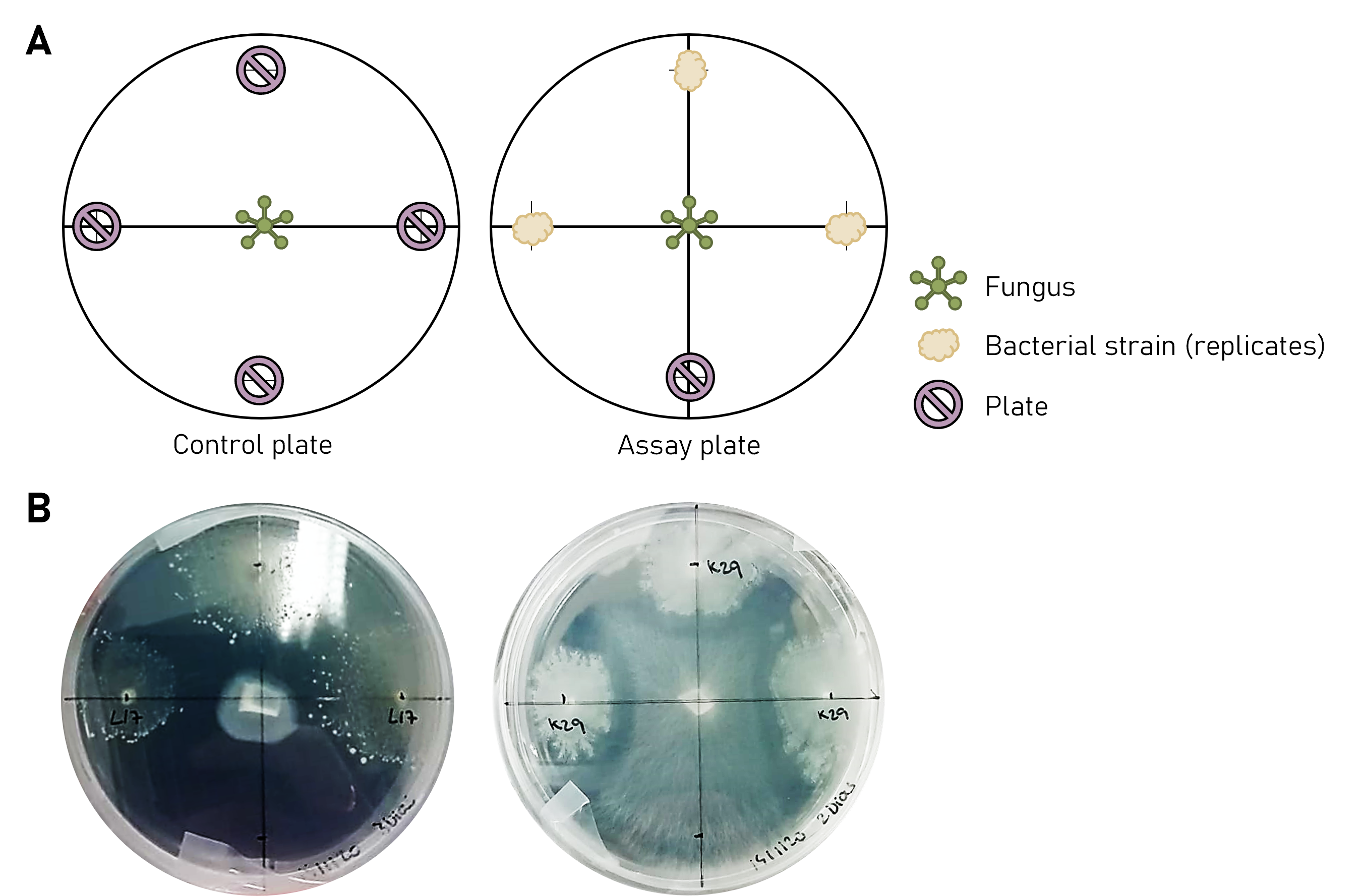

Supplement: Supplementary file 1 [file microorganisms-11-00057-s001.zip › Figure S2.tif]
